# Association of COVID-19 Vaccine Intake with Diagnosis, Hospitalization, and Oxygenation/Ventilation: A Longitudinal Analysis, 2021–2022, Japan

**DOI:** 10.3390/vaccines12111264

**Published:** 2024-11-08

**Authors:** Satomi Odani, Hitoshi Honda, Takahiro Tabuchi

**Affiliations:** 1 The Tokyo Foundation for Policy Research, Tokyo 106-6234, Japan; satomi.ichikawa1029@gmail.com; 2 Department of Infectious Diseases, School of Medicine, Fujita Health University, Toyoake 470-1192, Japan; hhhhonda@gmail.com; 3 Division of Epidemiology, School of Public Health, Tohoku University Graduate School of Medicine, Sendai 980-8575, Japan

**Keywords:** COVID-19 vaccine, Internet-based survey, longitudinal analysis, public health

## Abstract

**Background/Objectives:** Japan’s COVID-19 vaccination campaign achieved high coverage by 2022, yet limited national-level data has hindered evaluations of vaccine effectiveness. This study analyzed the impact of vaccines on infection outcomes while considering socioeconomic and behavioral factors in the Japanese population. **Methods:** A total of 19,482 individuals aged 16–81 years, who participated in both the 2021 (baseline) and 2022 (follow-up) waves of an Internet-based survey, were analyzed. Vaccine intake during the follow-up period (0/1/2+ doses) served as the exposure, while outcomes included COVID-19 diagnosis, hospitalization, and receipt of oxygenation/ventilation. Adjusted prevalence ratios (APRs) were calculated using Poisson regression models, controlling for baseline characteristics such as vaccination status, infection history, underlying medical conditions, socioeconomic factors, and preventive behaviors. **Results:** Overall, 81.6% of respondents received at least 1 dose of COVID-19 vaccine during the follow-up period. Among those without COVID-19 history at baseline (N = 19,182), 10.9% were diagnosed with COVID-19 in the past year, and 6.6% in the past 2 months. Respondents who received 1 or 2+ doses had lower diagnosis rates (APR = 0.76 and 0.43, respectively). For the past 2 months, only those with 2+ doses showed a significant reduction (APR = 0.51). Among 1999 diagnosed cases, those with 1 or 2+ doses showed lower hospitalization and oxygenation/ventilation likelihoods, though these differences were not statistically significant. **Conclusions:** The results supported the protective effect of COVID-19 vaccines against infection. Continued research is essential to further clarify the complex influence of vaccination, individual characteristics, and preventive behaviors on COVID-19 morbidity at the population level.

## 1. Introduction

The COVID-19 pandemic has posed significant challenges to public health systems worldwide, necessitating the development and implementation of effective vaccination strategies. In Japan, the government launched a national COVID-19 vaccination campaign in February 2021, initially targeting high-priority groups such as healthcare professionals, rescue workers, and public health center employees [1]. Subsequently, the campaign expanded to include individuals aged 65 or older in April 2021 and the general population with specific priority given to those with underlying health conditions [1]. This vaccination campaign progressed rapidly, with over 70% of Japanese residents having completed the recommended two-dose immunization course by the end of October 2021 [2]. To further enhance protection against COVID-19, booster doses were introduced in December 2021 [1]. The booster rollout aimed to provide an additional layer of immunity, particularly for individuals who had completed the primary vaccination series. By the end of May 2022, approximately 60% of the entire population had received a third vaccine dose [2]. These vaccination efforts have played a crucial role in mitigating the impact of the pandemic and reducing the incidence of COVID-19 in Japan.

Assessing the effectiveness of the COVID-19 vaccines and understanding the factors associated with vaccine uptake and their impact on disease outcomes require comprehensive data sources. In Japan, however, medical care information is maintained within the health insurance system, while the vaccination campaign is administered through a separate system governed by the Immunization Act. Moreover, the use of governmental vaccination records for research purposes is not permitted. The absence of a data linkage platform at the national level has posed challenges for evaluating the vaccine effectiveness and its interplay with individual characteristics, including demographic, socioeconomic, clinical, and behavioral characteristics.

A few case-control studies and subnational-level population-based studies have been conducted to investigate the efficacy of COVID-19 vaccines in the Japanese population [3,4,5,6,7,8]. Findings from these previous studies consistently showed high effectiveness in reduction in overall incidence of COVID-19 and the severity of illness due to COVID-19, over 80% for individuals fully vaccinated with two doses of the vaccine during the time when the delta variant was dominant [5,6,7]. During the omicron-dominant period, although the vaccine efficacy was reported to be lower than that during the delta-variant period, the estimates of vaccine effectiveness against infection ranged from 57% to 74% for those who received the booster [3,6].

Although these studies provided consistent results regarding vaccine efficacy, it is important to conduct a comprehensive assessment that takes into account the unique population characteristics in Japan on a large scale. Given that preventive behaviors such as wearing masks and avoiding risky situations were commonly practiced in Japan, understanding the interplay of these factors and their impact on promoting vaccination and preventing infections is crucial. Therefore, this study aims to contribute to the existing knowledge by providing evidence on the effectiveness of COVID-19 vaccines and investigating the associations between individual characteristics, vaccination, and infection outcomes in Japan. We considered vaccine intake as an individual’s practice of a preventive measure and took into account the practice of other preventive behaviors, as well as a variety of individual characteristics, allowing us to explore a broader perspective on the impact of preventive measures during the observed period.

## 2. Materials and Methods

### 2.1. Data

**Figure 1:**
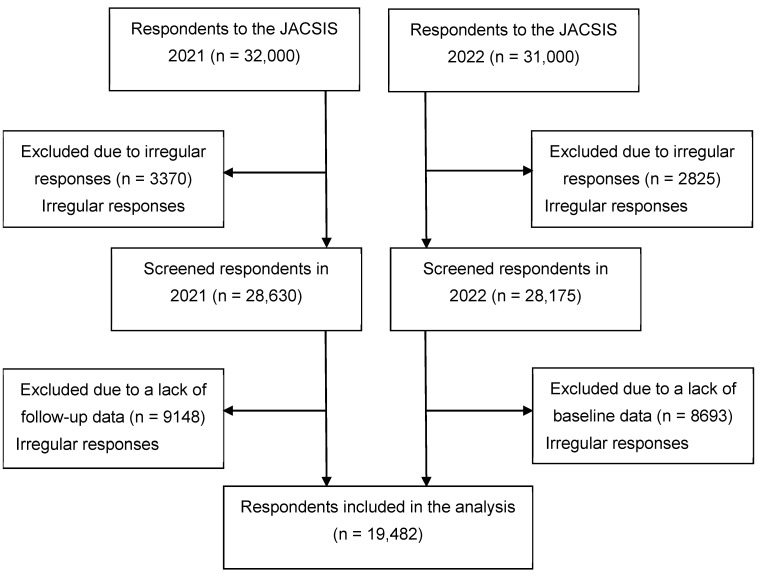
Flow diagram for respondent inclusion in the analysis, the Japan COVID-19 and Society Internet Survey (JACSIS), 2021–2022.

This study involved a longitudinal analysis of the 2021 and 2022 waves from the Japan COVID-19 and Society Internet Survey (JACSIS), a nationwide, Internet-based, self-reported survey targeting individuals aged 15 years or older. The initial JACSIS survey was conducted during August and September 2020, using a private vendor, Rakuten Insight Inc., which has 2.3 million panelists [9]. Participants were randomly selected from various demographic (including age, sex, and place of residence) and socioeconomic subgroups (including education, housing, and marital status), as defined by the Japan census. All participants were required to provide online informed consent [10]. Annual follow-up surveys were administered during August–September in 2021 and 2022, with sample replenishment. The survey period coincided with the end of the fifth wave of the COVID-19 epidemic (1 July –30 September 2021), driven by the delta variant, and the sixth (1 January–31 March 2022) and seventh waves (1 July–30 September 2022) in which the omicron variant was dominant. A total of 32,000 and 31,000 individuals responded to the 2021 and 2022 waves, respectively. From this pool, we excluded 3370 and 2825 individuals who provided irregular answers (from the 2021 and 2022 respondents, respectively) using a set of predefined questions incorporated into the questionnaire [10]. For example, individuals who responded to all multiple-choice items for illegal substance use (7 items) or presence of chronic conditions (15 items), those who answered with the same number over an entire set of questions, or those who chose a wrong answer for the question “Choose the second item from the bottom” were excluded. Ultimately, the analysis included 19,482 individuals who responded to both waves. The selection process of the analytical sample is depicted in Figure 1. The questionnaire used in the JACSIS survey is publicly available for download [11]. The Research Ethics Committee of the Osaka International Cancer Institute approved this study (no. 20084-9).

### 2.2. Exposure

The exposure variable in this analysis was the COVID-19 vaccine intake during the observation window. We calculated the difference in the number of completed doses between baseline (2021) and the 1-year follow-up (2022) for each participant. The doses were categorized as 0, 1, or 2+ based on this calculation. At baseline, respondents were asked to indicate their COVID-19 vaccine status using the following response choices: “Received 2 doses”, “Received 1 dose (plan to receive the second dose)/(do not plan to receive the second dose)/(received a 1-dose type vaccine)”, “Never received vaccine (cannot get a vaccine due to allergies or other health conditions)/(want to get a vaccine/already have an appointment for a vaccine)/(prefer to wait)/(do not want to get a vaccine)”. At the 1-year follow-up, respondents were asked the same question with the following response categories: “Received 4 doses/3 doses/2 doses/1 dose” and “Never received vaccine (cannot get a vaccine due to allergies or other health conditions)/(prefer to wait)/(do not need to get a vaccine)/(do not want to get a vaccine)”. We did not collect specific information on the type of vaccine each participant received, but given the national vaccine distribution strategy at the time of data collection, the majority of vaccinated individuals in our study likely received either the Pfizer-BioNTech or Moderna vaccines [12].

### 2.3. Outcome

We assessed three outcomes: COVID-19 diagnosis, COVID-19-induced hospitalization, and receipt of oxygen supplementation (referred to as “oxygenation” hereinafter) and/or mechanical ventilation (referred to as “ventilation” hereinafter) during hospitalization. COVID-19 infection was assessed by asking participants whether and when (in the past 2 months, 2 months to 1 year ago, or more than 1 year ago) they were diagnosed with COVID-19. We created dichotomous variables (yes/no) for the past-year infection and past-2-month infection separately. Participants were also asked with separate questions whether they were admitted to a hospital and received oxygenation and/or ventilation due to COVID-19 infection. We created dichotomous variables (yes/no) for past-year hospitalization and receipt of oxygenation and/or ventilation among hospitalized individuals.

### 2.4. Independent Variables

The independent variables assessed in this study included sex, age, education, employment, presence of underlying conditions (chronic respiratory illness, cardiac disease, kidney disease, cancer, diabetes, hypertension, and body mass index ≥ 30), smoking status, current use of heated tobacco products (HTPs), alcohol drinking, fear of COVID-19-induced death (yes/no) [13], and COVID-19 preventive behaviors. Participants were asked whether they wore a mask when other people were present, with response options of “always” or “sometimes/rarely/never”. Preventive behavior was further assessed regarding the avoidance of the “three Cs” (closed spaces, crowded places, and close-contact settings) which was recommended by the Japanese government [14]. Separate questions were asked for each “C”, and the number of times participants answered “always” (vs. “sometimes/rarely/never”) was summed and categorized into 0, 1, 2, or 3. Vaccination status at baseline was assessed as the number of doses that had been received before/at baseline (0/1/2).

### 2.5. Statistical Analysis

To account for potential selection bias of the Internet-based sample and non-response bias, we applied inverse probability weighting (IPW) to weight the data. Logistic regression models were fitted to compute propensity scores for “being an Internet survey respondent” using a nationally representative sample from the Comprehensive Survey of Living Conditions [15] as the reference. We controlled for demographic, socioeconomic, and behavioral characteristics (e.g., sex, age, residing region, marital status, education, employment, health status, tobacco product use) in the propensity score calculation. All analyses were weighted. Additional details regarding the IPW method are reported elsewhere [10,16].

We employed multivariable Poisson regression to investigate factors associated with COVID-19 vaccine intake during the observation window. Furthermore, we analyzed the associations between vaccine intake and COVID-19 infection among participants with no history of COVID-19 at baseline (N = 19,182), as well as the associations with hospitalization and receipt of oxygenation and/or ventilation among those diagnosed with COVID-19 during the observation window (N = 1999). Adjusted prevalence ratios (APRs) and 95% confidence intervals (CIs) were estimated, controlling for the aforementioned independent variables. These variables were either identified in the univariate analysis with a significance level of *p* < 0.1 or were deemed to have clinical and behavioral relevance in the context of vaccine intake. We assessed multicollinearity among independent variables using variance inflation factors, which were confirmed to be below 10. All analyses were performed using R version 4.2.2.

## 3. Results

Table 1 presents the baseline characteristics of the respondents and factors associated with COVID-19 vaccine intake during the observation window (N = 19,482). Overall, the majority of respondents (72.9%) had received two doses of the COVID-19 vaccine at baseline. Further, 90.9% reported always wearing a mask when in the presence of other people, 27.4% reported always avoiding all of the “three Cs” (closed spaces, crowded places, and close-contact settings), and 76.5% partially practiced the avoidance of the “three Cs”. Additionally, 40.1% reported a fear of death from COVID-19 and 81.6% reported receiving at least one dose of the COVID-19 vaccine between baseline and the 1-year follow-up, with 38.4%, 40.2%, 2.9%, and 0.1% having received one, two, three, and four doses, respectively. At follow-up, while a majority of respondents (76.1%) had completed booster vaccination (received 3+ doses), 11.6% had no vaccination history, and 12.2% had received one or two doses. The most significant association with COVID-19 vaccine intake during the observation window was seen with the baseline vaccine status. Those who had received one or two doses before/at baseline were more likely to receive additional doses (APR = 2.64, 95% CI = 2.45–2.84; APR = 2.38, 95% CI = 2.21–2.56) than those without vaccination history. Other groups that had a higher likelihood of vaccine intake during the observation window included those with a fear of COVID-19-induced death (APR = 1.05, 95% CI = 1.03–1.07) compared to those without the fear, those with underlying health conditions (APR = 1.03, 95% CI = 1.01–1.05) compared to those without them, current (past 30-day) alcohol drinkers (APR = 1.03, 95% CI = 1.01–1.05) compared to non-current/never drinkers, the elderly aged 65+ years (APR = 1.07, 95% CI = 1.05–1.10) compared to younger individuals, and self-employed (APR = 1.03, 95% CI = 1.002–1.06) and unemployed (APR = 1.03, 95% CI = 1.004–1.06) individuals compared to full-time workers. Groups with a lower likelihood of vaccine intake included those who did not avoid any of the “three Cs” (APR = 0.97, 95% CI = 0.94–0.99) and those who partially practiced the measure (avoided two of the “three Cs”) (APR = 0.97, 95% CI = 0.95–0.99) compared to those who avoided all of the “three Cs”, current smokers (APR = 0.96, 95% CI = 0.93–0.99) compared to never smokers, and part-time workers (APR = 0.95, 95% CI = 0.92–0.99) compared to full-time workers.

Table 2 presents the association between COVID-19 vaccine intake during the observation window and COVID-19 diagnosis among those with no COVID-19 history at baseline (N = 19,182). At the 1-year follow-up, 10.9% (N = 1999) and 6.6% (1192) reported having been diagnosed with COVID-19 in the past year and in the past 2 months. Past-year infection was significantly less likely among those who received one or 2+ doses of the COVID-19 vaccine during follow-up (APR = 0.76, 95% CI = 0.60–0.97; APR = 0.43, 95% CI = 0.34–0.55, respectively) compared to those who did not receive a vaccine. In particular, receipt of 2+ vaccine doses was significantly associated with reduced likelihood of COVID-19 diagnosis in the past 2 months (APR = 0.51, 95% CI = 0.36–0.71). Self-employed and unemployed individuals were also less likely to report past-year COVID-19 diagnosis (APR = 0.52, 95% CI = 0.39–0.70; APR = 0.52, 95% CI = 0.42–0.64, respectively) and the past-2-month diagnosis of COVID-19 (APR = 0.54, 95% CI = 0.38–0.78; APR = 0.58, 95% CI = 0.45–0.74, respectively) than full-time workers. Groups with a higher likelihood of COVID-19 infection were those who had received one dose of the COVID-19 vaccine at baseline (APR = 1.67, 95% CI = 1.21–2.28 for past-year infection) compared to those without vaccination, those with underlying health conditions (APR = 1.23, 95% CI = 1.05–1.43 for past-year infection) compared to those without them, and former smokers (APR = 1.26, 95% CI = 1.02–1.56 for past-2-month infection) compared to never smokers.

Table 3 presents the association of COVID-19 vaccine intake with hospitalization and the receipt of oxygenation and/or ventilation among those who were diagnosed with COVID-19 during the observation window (N = 1999). Of 1999 respondents, 10.1% (N = 195) and 7.0% (N = 131) of them reported hospitalization and receipt of oxygenation and/or ventilation during the 1-year observation window, respectively. Although it did not reach statistical significance, decreased likelihoods of hospitalization and oxygenation and/or ventilation were observed among those who received 1 or 2+ doses of the COVID-19 vaccine during follow-up; the APRs of hospitalization were 0.78 (95% CI = 0.42–1.44) and 0.87 (95% CI = 0.47–1.61), respectively, and those of oxygenation and/or ventilation were 0.86 (95% CI = 0.39–1.90) and 0.61 (95% CI = 0.27–1.36), respectively. For hospitalization, the only significant association was observed among males, with a 2.69 (95% CI = 1.59–4.55) times higher likelihood than females. Similarly, male sex was the strongest factor for oxygenation and/or ventilation (APR = 3.16, 95% CI = 0.62–6.17 vs. females), followed by age 65+ years (APR = 2.25, 95% CI = 1.01–4.99 vs. younger age) and the presence of underlying health conditions (APR = 1.70, 95% CI = 1.03–2.80 vs. non-presence). Individuals who reported current alcohol drinking were less likely to receive oxygenation and/or ventilation (APR = 0.56, 95% CI = 0.31–0.99 vs. those who did not).

## 4. Discussion

The intake of the COVID-19 vaccine was found to be significantly associated with a lower likelihood of COVID-19 diagnosis during the 1-year observation window. This period coincided with the seventh wave of the epidemic in Japan (1 July–30 September 2022), which resulted in the highest number of cases and deaths up to the time of data collection. As the first longitudinal study analyzing the interplay between population characteristics, vaccination, and infection outcomes in a large cohort in Japan, our findings contribute to the existing literature on COVID-19 vaccine effectiveness.

A noteworthy characteristic of our study is the high compliance rate among respondents with recommended preventive behaviors, such as mask-wearing and avoiding risky settings (the “three Cs”). This high compliance with government recommendations is a unique behavioral characteristic of the Japanese population during the COVID-19 epidemic. Studies have reported the effect of mask-wearing in preventing self-infection and reducing community transmission, even from asymptomatic individuals when it was implemented with other non-pharmaceutical control measures [17,18,19,20,21]. Avoidance of the “three Cs” evaluated in this study can be considered a form of social distancing, which has also been shown to delay or flatten the epidemic curve and consequently avert new COVID-19 and critical illness, even with modest reductions in contact among adults [19,20,21,22]. While these preventive behaviors may serve as an effective measure for population-level infection control, our study did not verify the specific effectiveness of these preventive behaviors for individuals, as various factors—such as the quality and type of masks used, consistency in adherence to social distancing, and situational challenges—may have confounded results.

Furthermore, we observed disparities in COVID-19 diagnosis rates based on employment status, with self-employed and unemployed individuals displaying lower infection rates. This trend may stem from reduced opportunities for close contact with others, as these groups often engage in less commuting and have fewer interactions in shared workspaces. This contrasts with full-time or part-time workers, who may have higher exposure rates. According to a survey by the Ministry of Internal Affairs and Communications, the implementation of remote work varied by industry during the COVID-19 pandemic, with high adoption rates in information and communication (55.7%), academic research and professional/technical services (43.2%), and finance and insurance (30.2%) [23]. In contrast, sectors like healthcare (4.3%) and accommodation (11.1%) had notably lower remote-work rates [23], suggesting that employees in these fields faced greater risks of COVID-19 infection.

Our findings also revealed that individuals with underlying medical conditions had a higher likelihood of infection. This increase may be attributable to these individuals having more frequent clinical visits and being more likely to undergo COVID-19 testing, as many healthcare facilities routinely tested patients presenting with symptoms like fever. We assessed hospitalization and receipt of oxygenation and/or ventilation as indicators of severe illness related to COVID-19. However, in Japan, the decision to hospitalize patients can vary significantly based on the capacity of healthcare facilities, which was particularly strained during the peak of the seventh wave [24]. This variability complicates our ability to interpret hospitalization rates accurately. Among study participants, underlying medical conditions were the only factor consistently associated with hospitalization. Receipt of oxygenation or ventilation was significantly linked to underlying conditions, male sex, and age over 65, corroborating known risk factors for severe COVID-19 progression [25]. We also found that individuals who consumed alcohol had a lower likelihood of requiring oxygenation or ventilation. This finding may suggest that alcohol consumption is linked to various demographic factors, yet our analysis did not reveal significant differences between drinkers and non-drinkers regarding the known risk factors. Thus, the observed negative association might reflect unmeasured biases in our sample or the relatively rare nature of the outcome event.

This study has several limitations. First, we were unable to establish a causal relationship due to the lack of information on the chronological order of vaccine intake and the outcomes. Nevertheless, a significant portion of respondents likely completed their vaccinations prior to the peak of the seventh wave, as indicated by national vaccination rates with over 70% having received the two-dose regimen and approximately 60% having received a third dose by the end of June 2022 [2]. Second, our analysis did not capture potential behavioral changes following vaccination or infection. For instance, individuals vaccinated may have engaged in riskier behaviors, believing themselves to be protected, or those who contracted COVID-19 may have ceased vaccination due to perceived ineffectiveness. However, our results indicated that prior COVID-19 history did not affect subsequent vaccination during the study period. Third, respondents who died or experienced severe illness from COVID-19 may have been underrepresented in the follow-up survey, though Japan’s relatively low mortality rate diminishes concerns about significant bias. Fourth, the self-reported nature of the survey might have led to recall bias and misunderstanding of the questions. Fifth, as our sample was collected through Internet-based recruitment, our findings may not be generalizable to populations with limited Internet access or literacy. However, over 90% of the Japanese population had access to the Internet as of 2021 [26], and this study used weighted data to address differences in key socioeconomic and demographic characteristics and tobacco use behavior between the respondents of this Internet survey and a nationally representative population. Lastly, there may be other factors not assessed in this study that contributed to the outcomes. Specifically, this study did not consider the type of COVID-19 vaccine or detailed patterns of preventive and risky behaviors of the respondents. Further research is needed to elucidate the interaction of these factors and their effect on the outcomes in real-world settings.

## 5. Conclusions

Considering demographic, socioeconomic, medical, and behavioral characteristics, COVID-19 vaccination was linked to a significantly lower likelihood of COVID-19 diagnosis, while other assessed preventive measures—such as mask-wearing and social distancing—did not show significant associations with lower diagnosis likelihood at the individual level during Japan’s peak period of COVID-19 cases and fatalities. Continued assessment of vaccine efficacy and effectiveness is essential to inform future strategies that benefit public health and society.

## Figures and Tables

**Table 1:** Baseline characteristics and 1-year vaccine intake, 2021–2022, Japan.

	Distribution	Received 1+ Dose of the COVID-19 Vaccine During the 1-Year Observation Window	
Baseline Characteristics	N (%)	% (SE)	APR (95% CI)
Total	19,482 (100.0%)	81.6 (0.5)	-
Vaccination status (number of COVID-19 vaccine doses completed before/at baseline)			
0	3155 (18.1%)	36.5 (1.4)	Ref.
1	1579 (9.0%)	97.4 (0.8)	**2.64 (2.45–2.84)**
2	14,748 (72.9%)	91.0 (0.4)	**2.38 (2.21–2.56)**
Mask-wearing			
No	1558 (9.1%)	67.5 (2.0)	0.95 (0.90–1.001)
Yes	17,924 (90.9%)	83.0 (0.5)	Ref.
Avoidance of risky situations (number of the “three Cs” avoided)			
0	4162 (23.5%)	76.1 (1.1)	**0.97 (0.94–0.99)**
1	4399 (22.8%)	81.0 (0.9)	0.98 (0.95–1.002)
2	5221 (26.2%)	82.9 (0.9)	**0.97 (0.95–0.99)**
3	5700 (27.4%)	85.6 (0.7)	Ref.
Fear of COVID-19-induced death			
No	11,780 (59.9%)	78.8 (0.6)	Ref.
Yes	7702 (40.1%)	85.8 (0.6)	**1.05 (1.03–1.07**)
Underlying medical conditions			
Not present	13,166 (67.1%)	79.1 (0.6)	Ref.
Present	6316 (32.9%)	86.8 (0.8)	**1.03 (1.01–1.05**)
Ever diagnosed with COVID-19			
No	19,182 (98.3%)	81.7 (0.5)	Ref.
Yes	300 (1.7%)	76.3 (4.4)	1.01 (0.90–1.14)
Smoking status			
Never	10,667 (55.8%)	80.9 (0.6)	Ref.
Former	6023 (28.7%)	85.1 (0.8)	0.99 (0.97–1.01)
Current	2792 (15.4%)	77.6 (1.2)	**0.96 (0.93–0.99)**
Current use of heated tobacco products			
No	17,817 (90.6%)	81.6 (0.5)	Ref.
Yes	1665 (9.4%)	81.7 (1.5)	1.02 (0.98–1.06)
Current alcohol drinking			
No	9173 (62.0%)	80.0 (0.7)	Ref.
Yes	10,309 (38.0%)	84.2 (0.6)	**1.03 (1.01–1.05)**
Sex			
Female	9751 (50.5%)	81.7 (0.7)	Ref.
Male	9731 (49.5%)	81.5 (0.6)	1.01 (0.99–1.03)
Age, years old			
16–64	13,870 (72.6%)	78.0 (0.6)	Ref.
65+	5612 (27.4%)	91.1 (0.7)	**1.07 (1.05–1.10)**
Education			
Some college/college or higher	13,875 (47.8%)	81.9 (0.5)	Ref.
High school or less	5506 (52.2%)	81.6 (0.7)	0.99 (0.97–1.01)
Employment status			
Full time	7199 (35.8%)	82.2 (0.7)	Ref.
Self-employed	1384 (6.7%)	72.5 (2.0)	**1.03 (1.002–1.06)**
Part time	3798 (21.1%)	80.3 (1.0)	**0.95 (0.92–0.99)**
Unemployed	7101 (36.4%)	83.5 (0.8)	**1.03 (1.004–1.06)**

Abbreviations: APR = adjusted prevalence ratio, CI = confidence interval, COVID-19 = coronavirus disease 2019, three Cs = closed spaces, crowded places, and close-contact settings, SE = standard error. Note: Data were extracted from the Japan COVID-19 and Society Internet Survey (JACSIS), a nationwide, self-reported survey and were weighted to account for the selectivity bias of the Internet-based sample using a nationally representative sample as the reference. APRs and CIs were computed by multivariable Poisson regression analysis.

**Table 2:** Percentage and adjusted ratio of COVID-19 diagnosis among infection-naïve individuals, 2021–2022, Japan.

	Distribution	COVID-19 Diagnosis During the 1-Year Observation Window		COVID-19 Diagnosis in the Past 2 Months	
Characteristics	N (%)	% (SE)	APR (95% CI)	% (SE)	APR (95% CI)
Total	19,182 (100.0%)	10.9 (0.4)	-	6.6 (0.3)	-
1-year COVID-19 vaccine intake (number of doses received during follow-up)					
0	3130 (18.3%)	14.7 (1.0)	Ref.	8.2 (0.8)	Ref.
1	7475 (39.3%)	13.5 (0.7)	**0.76 (0.60–0.97)**	8.4 (0.5)	0.87 (0.62–1.23)
2+	8419 (42.4%)	6.5 (0.5)	**0.43 (0.34–0.55)**	4.3 (0.4)	**0.51 (0.36–0.71**)
Vaccination status at baseline (number of completed doses of the COVID-19 vaccine)					
0	3065 (18.0%)	11.2 (0.9)	Ref.	6.5 (0.7)	Ref.
1	1497 (8.5%)	15.6 (1.5)	**1.67 (1.21–2.28)**	9.3 (1.3)	1.53 (0.99–2.35)
2	14,620 (73.4%)	10.2 (0.4)	1.05 (0.81–1.36)	6.3 (0.3)	1.04 (0.72–1.50)
Mask-wearing					
No	1440 (8.5%)	14.9 (1.6)	0.91 (0.72–1.15)	7.8 (1.3)	1.07 (0.77–1.50)
Yes	17,742 (91.5%)	10.5 (0.4)	Ref.	6.5 (0.3)	Ref.
Avoidance of risky situations (number of the “three Cs” avoided)					
0	4024 (23.1%)	13.0 (0.9)	1.04 (0.86–1.27)	7.7 (0.7)	1.11 (0.86–1.43)
1	4331 (22.8%)	12.0 (0.8)	1.13 (0.93–1.37)	7.5 (0.7)	1.23 (0.96–1.58)
2	5172 (26.5%)	9.0 (0.6)	0.93 (0.77–1.12)	6.0 (0.5)	1.05 (0.83–1.33)
3	5655 (27.7%)	9.8 (0.7)	Ref.	5.6 (0.5)	Ref.
Fear of COVID-19-induced death					
No	11,575 (59.8%)	11.1 (0.5)	Ref.	7.0 (0.4)	Ref.
Yes	7607 (40.2%)	10.5 (0.6)	1.00 (0.87–1.15)	6.1 (0.4)	0.89 (0.75–1.07)
Underlying medical conditions					
Not present	13,024 (67.5%)	11.1 (0.4)	Ref.	6.6 (0.3)	Ref.
Present	6158 (32.5%)	10.3 (0.7)	**1.23 (1.05–1.43)**	6.6 (0.6)	1.23 (0.99–1.52)
Smoking status					
Never	10,555 (56.2%)	10.2 (0.5)	Ref.	5.9 (0.4)	Ref.
Former	5932 (28.6%)	12.1 (0.7)	1.16 (0.98–1.36)	8.0 (0.6)	**1.26 (1.02–1.56)**
Current	2695 (15.2%)	10.9 (1.0)	0.83 (0.65–1.05)	6.9 (0.8)	0.86 (0.64–1.16)
Current HTP use					
No	17,618 (91.0%)	10.3 (0.4)	Ref.	6.3 (0.3)	Ref.
Yes	1564 (9.0%)	16.1 (1.8)	1.25 (0.97–1.63)	10.1 (1.3)	1.26 (0.95–1.67)
Current alcohol drinking					
No	9009 (62.0%)	10.0 (0.5)	Ref.	5.9 (0.4)	Ref.
Yes	10,173 (38.0%)	12.3 (0.5)	1.07 (0.94–1.22)	7.7 (0.5)	1.11 (0.93–1.32)
Sex					
Female	9651 (50.9%)	9.7 (0.5)	Ref.	5.6 (0.4)	Ref.
Male	9531 (49.1%)	12.1 (0.5)	1.04 (0.89–1.22)	7.7 (0.5)	1.16 (0.95–1.41)
Age, years					
16–64	13,590 (72.2%)	13.1 (0.5)	Ref.	7.8 (0.4)	Ref.
65+	5592 (27.8%)	5.2 (0.6)	0.80 (0.60–1.07)	3.5 (0.6)	0.80 (0.54–1.17)
Education					
Some college/college or higher	13,651 (47.7%)	12.1 (0.5)	Ref.	6.9 (0.3)	Ref.
High school or less	5434 (52.3%)	9.7 (0.6)	0.89 (0.78–1.02)	6.4 (0.5)	1.01 (0.85–1.21)
Employment status					
Full time	7030 (35.5%)	15.3 (0.7)	Ref.	9.1 (0.5)	Ref.
Self-employed	1356 (6.7%)	7.1 (1.0)	**0.52 (0.39–0.70)**	4.5 (0.8)	**0.54 (0.38–0.78)**
Part time	3746 (21.1%)	13.4 (0.9)	0.97 (0.82–1.16)	8.1 (0.8)	1.02 (0.81–1.29)
Unemployed	7050 (36.7%)	5.8 (0.5)	**0.52 (0.42–0.64)**	3.7 (0.4)	**0.58 (0.45–0.74)**

Abbreviations: APR = adjusted prevalence ratio, CI = confidence interval, COVID-19 = coronavirus disease 2019, three Cs = closed spaces, crowded places, and close-contact settings, SE = standard error. Note: Data were extracted from the Japan COVID-19 and Society Internet Survey (JACSIS), a nationwide, self-reported survey and were weighted to account for the selectivity bias of the Internet-based sample using a nationally representative sample as the reference. APRs and CIs were computed by multivariable Poisson regression analysis.

**Table 3:** Percentage and adjusted ratio of hospitalization and receipt of oxygenation among infected individuals, 2021–2022, Japan.

	Distribution	Hospital Admission		Hospital Admission + Oxygenation and/or Ventilation	
Baseline Characteristics	N (%)	% (SE)	APR (95% CI)	% (SE)	APR (95% CI)
Total	1999 (100.0%)	10.1 (1.2)	-	7.0 (1.0)	-
1-year COVID-19 vaccine intake (number of doses received during follow-up)					
0	473 (25.1%)	10.4 (2.1)	Ref.	8.0 (1.7)	Ref.
1	998 (49.3%)	7.9 (1.4)	0.78 (0.42–1.44)	5.5 (1.3)	0.86 (0.39–1.90)
2+	489 (25.6%)	10.6 (2.5)	0.87 (0.47–1.61)	5.5 (1.8)	0.61 (0.27–1.36)
Vaccination status at baseline (number of completed doses of the COVID-19 vaccine)					
0	365 (18.6%)	9.7 (2.2)	Ref.	8.2 (2.1)	Ref.
1	211 (12.3%)	12.2 (4.4)	1.12 (0.47–2.70)	10.0 (4.3)	1.04 (0.35–3.10)
2	1423 (69.1%)	9.8 (1.4)	0.99 (0.51–1.92)	6.2 (1.1)	0.69 (0.31–1.54)
Underlying medical conditions					
Not present	1434 (69.2%)	8.2 (1.1)	Ref.	5.1 (0.8)	Ref.
Present	565 (30.8%)	14.4 (2.7)	1.33 (0.83–2.13)	11.3 (2.6)	**1.70 (1.03–2.80)**
Smoking status					
Never	1103 (52.9%)	8.1 (1.3)	Ref.	5.8 (1.2)	Ref.
Former	648 (31.9%)	11.8 (2.3)	1.06 (0.64–1.77)	6.7 (1.8)	0.86 (0.46–1.62)
Current	248 (15.2%)	13.5 (3.6)	0.98 (0.57–1.71)	12.1 (3.5)	1.20 (0.65–2.21)
Current HTP use					
No	1783 (86.7%)	10.0 (1.3)	Ref.	7.0 (1.1)	Ref.
Yes	216 (13.3%)	10.8 (2.7)	1.11 (0.64–1.93)	7.5 (2.0)	1.10 (0.61–1.99)
Current alcohol drinking					
No	897 (57.0%)	9.7 (1.5)	Ref.	7.5 (1.3)	Ref.
Yes	1102 (43.0%)	10.6 (1.8)	0.75 (0.48–1.17)	6.4 (1.5)	**0.56 (0.31–0.99)**
Sex					
Female	962 (45.5%)	5.1 (1.0)	Ref.	3.2 (0.9)	Ref.
Male	1037 (54.5%)	14.3 (1.9)	**2.69 (1.59–4.55)**	10.2 (1.6)	**3.16 (1.62–6.17)**
Age, years					
16–64	1771 (86.8%)	9.3 (1.1)	Ref.	6.5 (1.0)	Ref.
65+	228 (13.2%)	15.5 (4.9)	1.85 (0.94–3.62)	10.4 (4.3)	**2.25 (1.01–4.99)**
Education					
Some college/college or higher	1483 (53.1%)	9.9 (1.3)	Ref.	7.0 (1.1)	Ref.
High school or less	503 (46.9%)	10.4 (2.0)	0.83 (0.52–1.32)	7.2 (1.8)	0.70 (0.38–1.30)

Abbreviations: APR = adjusted prevalence ratio, CI = confidence interval, COVID-19 = coronavirus disease 2019, SE = standard error. Note: Data were extracted from the Japan COVID-19 and Society Internet Survey (JACSIS), a nationwide, self-reported survey and were weighted to account for the selectivity bias of the Internet-based sample using a nationally representative sample as the reference. APRs and CIs were computed by multivariable Poisson regression analysis.

## Data Availability

The data are not publicly available due to privacy restrictions and are only available on request to the corresponding author at tabuchitak@gmail.com.

## Abbreviations

APR	Adjusted prevalence ratio
CI	Confidence interval
COVID-19	Coronavirus disease 2019
HTP	Heated tobacco product
IPW	Inverse probability weighting
JACSIS	Japan COVID-19 and Society Internet Survey
SARS-CoV-2	Severe acute respiratory syndrome coronavirus 2
